# Comparison of methods for correcting QT interval in athletes and young people: A systematic review

**DOI:** 10.1002/clc.24093

**Published:** 2023-07-20

**Authors:** Shehane Mahendran, Ishita Gupta, Jason Davis, Angus J. Davis, John W. Orchard, Jessica J. Orchard

**Affiliations:** ^1^ Sydney School of Public Health The University of Sydney Sydney Australia; ^2^ Royal Brisbane and Women's Hospital Brisbane Australia; ^3^ School of Medicine University of Queensland Brisbane Australia

**Keywords:** Bazett, electrocardiogram, Fridericia, QT interval correction, screening

## Abstract

Screening elite athletes for conditions associated with sudden cardiac death is recommended by numerous international guidelines. Current athlete electrocardiogram interpretation criteria recommend the Bazett formula (QTcB) for correcting QT interval. However, other formulae may perform better at lower and higher heart rates (HR). This review aimed to examine the literature on various QT correction methods in athletes and young people aged 14−35 years and determine the most accurate method of calculating QTc in this population. A systematic review of MEDLINE, EMBASE, Scopus, and SportDiscus was performed. Papers comparing at least two different methods of QT interval correction in athletes or young people were included. Quality and risk of bias were assessed using a standardized tool. The search strategy identified 545 papers, of which 10 met the criteria and were included. Nine of these studies concluded that QTcB was least reliable for removing the effect of HR and was inaccurate at both high (>90 beats per min [BPM]) and low (<60 BPM) HRs. No studies supported the use of QTcB in athletes and young people. Alternative QT correction algorithms such as Fridericia (QTcF) produce more accurate correction of QT interval at HRs seen in athletes and young people. QTcB is less accurate at lower and higher HRs. QTcF has been shown to be more accurate in these HR ranges and may be preferred to QTcB for QTc calculation in athletes and young people. However, accurate QTc reference values for discrete HRs using alternative algorithms are not well established and require further research.

## Graphical abstract

This systematic review aimed to determine which QT correction formulae is the most accurate in athletes and young people. Although Bazett is the most common, it has been found to be the least reliable. Fridericia has been shown to be the most accurate QT interval correction formula in these groups.

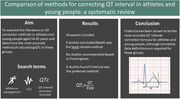

## 1 INTRODUCTION

Sudden cardiac death (SCD) is the most frequent cause of mortality in athletes during exercise, with an estimated incidence of approximately 0.46–3.7 events per 100 000 person years.^1^, ^2^ The majority of these events are a result of undiagnosed arrhythmias, channelopathies, and cardiomyopathies, many of which may be identified on a resting 12‐lead electrocardiogram (ECG).^2^, ^3^ Hence, preparticipation cardiac screening of athletes for conditions associated with SCD is recommended by international guidelines.^4^, ^5^

Long QT syndrome (LQTS) is a major cause of SCD from ventricular arrhythmias in athletes and young people.^6^ The prevalence is estimated to be approximately 1 in 2000 individuals, though the true rate is thought to be higher.^7^ Presentations of LQTS vary from asymptomatic individuals identified on screening or genetic testing, to syncope, seizures, resuscitated cardiac arrest, or sudden arrhythmogenic cardiac death. LQTS is one of the conditions that can be potentially identified as part of cardiac screening of athletes and is treatable, often allowing safe return to sport.

As the QT interval varies with heart rate (HR), it requires correction to a standardized HR of 60 beats per min (BPM) for interpretation.^4^ The most commonly used algorithm for QT correction is the Bazett method (QTcB), a logarithmic formula which was proposed in 1920,^8^ which is recommended by the International Criteria for ECG Interpretation in Athletes.^4^ QTcB has been utilized in almost all the diagnostic, prognostic, and treatment studies of LQTS to date.

**Figure 1:**
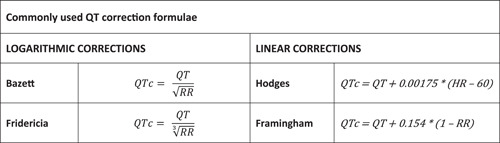
Commonly used QT correction formulae.

However, QTcB has known limitations at extreme HRs, particularly at HRs below 60 BPM and above 90 BPM, which is acknowledged in the International Criteria.^4^, ^9^ Other studies have suggested that QTcB is most effective at HRs between 50 and 70 BPM,^10^ and inappropriate at HRs above 80 BPM.^11^ This is particularly important in younger athletes, who may have a higher resting HR than adult athletes.^11^ Sinus bradycardia, defined as a HR less than 60 BPM, is present in up to 80% of highly trained athletes.^12^, ^13^ Further, QTcB has mainly been studied in an older adult population.^2^, ^14^ A number of other formulae have been proposed (Figure 1), such as Fridericia (QTcF) (also a logarithmic correction), Hodges (QTcH), and Framingham (QTcFr) (both linear corrections), which may perform better than QTcB at lower and higher HRs, and may be more accurate for athletes and young people.

Inaccurate QTc calculation may have important clinical implications. It could result in unnecessary follow‐up testing or even diagnostic errors with substantial implications for individuals who could be given an erroneous diagnosis of LQTS, or incorrectly provided reassurance that they are not affected. A method to account for QT interval variation by HR is to retest if the original ECG trace was outside the recommended range of 60−90 BPM.^4^ This is appropriate advice for patients in a long QT clinic, for example. The reality of some clinical situations, such as athlete screening, is that it may not be practical to instruct patients to rest or exercise and then have a repeat ECG at the desired HR. For the real world, a QT correction interval that works “first time” for all likely HRs that athletes and young people present with would be preferable.

Therefore, this study sought to perform a systematic literature review of QT interval correction methods in athletes and young people aged 14−35 years to determine which formula is the most accurate (i.e., best removes the effect of HR), especially at the low and high HRs commonly found in this population.

## 2 MATERIALS AND METHODS

The systematic review was performed according to Preferred Reporting Items for Systematic Reviews and Meta‐Analysis (PRISMA)^15^ and registered with the International Prospective Register of Systematic Reviews, PROSPERO on April 9, 2022 (ID CRD42022313190).

### 2.1 Search strategy

An initial search planner was formulated with key search terms, which were used individually and combined. Search terms included (athlete OR sportsperson OR young adult OR player) AND (ECG OR EKG OR QT OR QT interval). A full list of search terms and the search strategy is available in Supporting Information: Appendix A. The initial search was conducted on April 9, 2022 with the following databases searched: MEDLINE, EMBASE, Scopus, and SPORTDiscus. The search language was limited to English. There was no restriction on the publication date. A subsequent search was carried out with citation chaining of the included articles to identify any further potential articles. We also searched gray literature for articles that may not have been identified through the computer‐assisted search strategy. The latest update of the search was conducted in July 2022 and identified no additional relevant studies.

Articles were added to Covidence, and this software was used for screening and data collection. Potentially relevant articles were screened for eligibility independently in an unblinded standardized manner by two reviewers (I. G. and S. M.) based on title and abstract after the removal of duplicates. Any disagreements were resolved by a third author (J. D.) or by discussion with the remaining authors as required. Further screening by full text review of the articles identified through initial screening was conducted independently by three investigators (I. G., S. M., and J. D.), with each paper being reviewed by two reviewers, to determine whether they met the inclusion criteria. Discrepancies between the reviewers were resolved by discussion with the rest of the authors.

### 2.2 Study selection

Observational studies using different methods of QT interval correction in athletes or young people aged 14−35 years of age within an outpatient setting were selected. The age group was selected based on the European Society of Cardiology definitions of a young athlete being below the age of 35 years, and with the intention of excluding the pediatric population.^16^ Given the small number of studies in this area, we extended the age range to a minimum of 14 years to allow inclusion of a sufficient number of studies for analysis. We considered athletes as individuals who engage in exercise or training for sport or general fitness, with a premium on performance, often engaged in individual or team competition and estimated to exercise intensively for at least 4−8 h per week. Observational studies included cohort and cross‐sectional study designs.

Studies were included if they (i) compared at least two QT correction methods, (ii) analyzed an athlete population or people aged 14−35 years old, (iii) used outpatient, screening, or resting ECGs, and (iv) used a 12‐lead‐ECG. Only original research articles with an abstract written in English were included. If a paper had a title and abstract in English and was included as relevant during abstract screening, if the full text was in another language, the paper was translated into English with the assistance of Google Translate and underwent a full text review by all authors.

Studies were excluded if they: (i) included mainly non‐exercising individuals, (ii) included participants aged predominantly under 14 or over 35 years, (iii) analyzed patients within inpatient, or Emergency Department settings, (iv) used stress test ECGs or Holter studies, (v) involved acquired causes of LQTS (e.g., through medication use), (vi) assessed only one method of QT interval correction, or (vii) used the same data set as another included study. Other systematic reviews or meta‐analyses were also excluded. If there were multiple studies performed by the same group of authors using the same patient data set, only the more recent study was included. If studies included a minority of patients outside the included age range and they provided results by age, data were extracted from these studies to only include participants within our selected age range.

### 2.3 Data extraction

Data extraction fields were developed with all authors in agreement. These were pilot tested on a randomly selected included study and were refined over time. Data extraction was conducted independently by two authors (S. M. and I. G.), with a third author checking the extracted data (J. D.). Any discrepancies were resolved by discussion amongst all authors. The following information was extracted from each included paper: country where the study was conducted, study design, population, sample size, HR range of participants, characteristics of study participants (proportion of male participants, age [range and/or mean with standard deviation]), QT correction methods used, which QT correction methods were preferred and why, correlation coefficient (*R* ^2^) for each method, reference range, average QTc, and upper limits of QTc for each included formula. Data extraction was performed using Covidence software. The data extraction template is available in Supporting Information: Appendix B.

### 2.4 Quality and risk of bias assessment

Quality assessment and internal validity of the included studies were evaluated independently by two authors (S. M. and I. G.) based on the NHLBI Study Quality Assessment Tool.^17^ Each study was graded as “Good,” “Fair,” or “Poor” based on the attributes evaluated and risk of bias. Discrepancies were subsequently resolved by a third, independent author (J. D.).

### 2.5 Data synthesis and analysis

Positive and negative recommendations regarding various QTc correction methods, as well as other categories of data extraction as listed above, were summarized. Concordant data was extracted from Covidence in tabular format. Where available, *R* ^2^ values reported from each study were analyzed as a correction measure to remove the correlation between HR and QTc.

## 3 RESULTS

### 3.1 Search results

**Figure 2:**
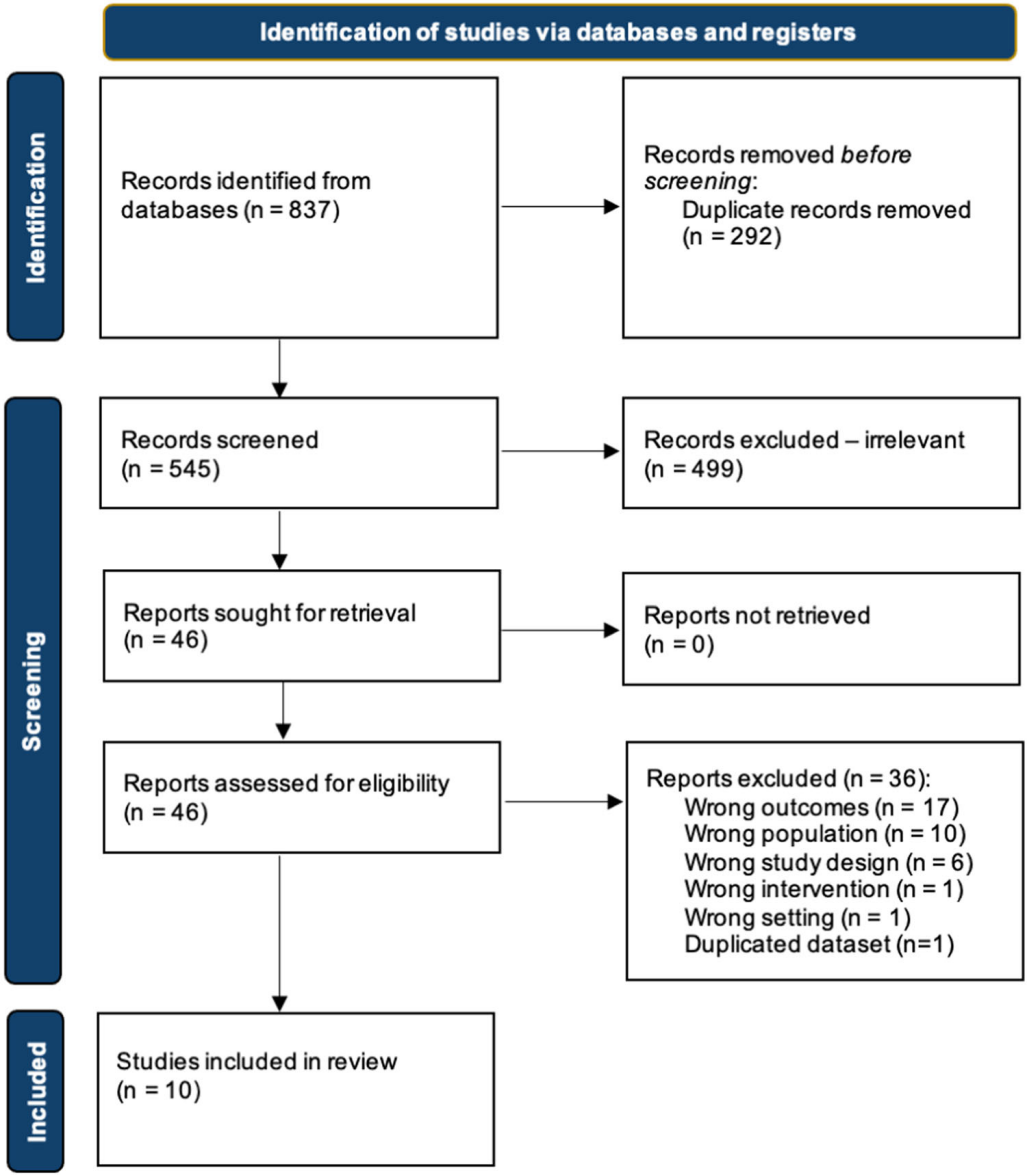
PRISMA (preferred reporting items for systematic reviews and meta‐analyses) flow chart for literature search and study selection.

The initial search strategy identified a total of 837 results from electronic databases. After duplicates (*n* = 292) were removed, there were 545 articles remaining for abstract and title screening. A total of 46 records were retrieved for full text evaluation, of which 10 studies met the eligibility criteria and were included for data extraction and synthesis.^18^, ^19^, ^20^, ^21^, ^22^, ^23^, ^24^, ^25^, ^26^, ^27^ Figure 2 outlines the study selection process.

### 3.2 Characteristics of the studies

Of the 10 included studies, 9 (90%) had a cross‐sectional design^18^, ^19^, ^20^, ^21^, ^22^, ^23^, ^25^, ^26^, ^27^ whilst the remaining 1 (10%) had a cohort design (Table 1).^24^ The cohort study was conducted in Slovakia in 2013. There were 2 studies published in the 1990s^18^, ^23^ and 8 published between 2012 and 2019.^20^ There were 44 501 participants included across all studies, of which 57.6% (*n* = 25, 621) were male. In most studies, the majority of participants were male (8 out of 10, with 3 of these 8 having a 100% male population). One study had a majority of female participants,^18^ and 1 study included a 100% female population.^24^ The mean age was provided for 5 studies and was within our age range of 14−35 years old.^19^, ^20^, ^21^, ^25^, ^26^ There were 5 studies for which mean age was not provided, however, for all 5 studies the age range was within 14−35 years old.^18^, ^22^, ^23^, ^24^, ^27^ Only 1 study was conducted outside of Europe or Northern America^18^ and 3 studies specifically included only Caucasian participants.^19^, ^24^, ^27^

**Table 1:** Characteristics of included studies.

First author, year	Country	Population	Sample size	Male (*n*, %)	Age range (years)	Mean age (SD)	Overall quality assessment	QT correction methods used
Aihoshi (1995)^18^	Japan	Young people	5,786	2741, 47.36%	15	15	Fair	Bazett Fridericia Others (not Framingham or Hodges)
Gervasi (2017)^19^	Italy	Athletes	373	373, 100%	13−35	14.3 (5.6)	Fair	Bazett Fridericia Framingham Hodges
Griffet (2016)^20^	France	Athletes	446	237, 53.1%	10−18	14.8 (1.8)	Fair	Bazett Fridericia Framingham Hodges
Hadley (2019)^21^	United States	Athletes and young people	31 588	17 318, 55%	12−35	16.9 (2.7)	Fair	Bazett Fridericia Framingham Hodges
Huttin (2018)^22^	France	Athletes	2484	2848, 100%	16−40	Not provided	Fair	Bazett Fridericia
Karjalainen (1994)^23^	Finland	Young people	324	324, 100%	18−28	Not provided	Fair	Bazett Fridericia Framingham
Kujanik (2013)^24^	Slovakia	Young people	138	0, 0%	18−24	Not provided	Fair	Bazett Fridericia Hodges
Oo (2016)^25^	United States	Young people	106	104, 98%	NR	29.7 (2.7)	Fair	Bazett Fridericia Framingham Hodges
Pickham (2016)^26^	United States	Athletes	2077	1288, 62%	14−35	19 (3.5)	Fair	Bazett Fridericia Framingham Hodges
Wong (2012)^27^	France	Athletes and young people	1179	752, 64%	17−38	NR	Fair	Bazett Fridericia Framingham Hodges Linear exponential regression

Abbreviations: NR, not reported; SD, standard deviation.

### 3.3 Quality and risk of bias assessment

Quality assessment judged all studies as “Fair.” No studies were excluded for quality. Details appear in Supporting Information: Appendix C.

### 3.4 Comparison of QT interval correction methods

All studies compared the Bazett and Fridericia formulae with at least one other QT correction formula. The other commonly included formulae were Hodges and Framingham. There were 7 studies that compared Framingham, 7 studies that compared Hodges, and 5 studies that compared one or more other formulae.

There were no studies that supported the use of QTcB within young adults and athletes (Table 2). Nine studies concluded that QTcB was least reliable method for removing the effect of HR and was inaccurate at both high and low HRs across all age ranges.^18^, ^19^, ^20^, ^21^, ^22^, ^23^, ^25^, ^26^, ^27^ These studies concluded that QTcB tends to overestimate QTc at higher HRs and underestimate at low HRs. Most of the included studies did not define the upper limit of HR at which QTcB formula becomes less reliable. Karlajainin et al. defined high HR as >99 BPM, while Kujanik et al. reported that QTcB should be used for the average physiological HR only but did not provide further cut offs, though the resting HR range of individuals in the study was 48−90 BPM.^23^, ^24^

**Table 2:** Results.

First author, year	HR range (BPM)	Mean HR (SD) (BPM)	*R* ^2^ value				Mean QTc (SD)				Recommendation
							Median QTc (95% CI)				
			QTcB	QTcF	QTcFr	QTcH	QTcB	QTcF	QTcFr	QTcH	
Aihoshi (1995)^18^	NR	NR	NR	NR	NR	NR	NR	NR	NR	NR	QTcF or an exponential formula
Gervasi (2017)*^19^	NR	66 (11) & 57 (10)	0.37 & 0.19	0.01 & 0.00	0.55 & 0.55	0.00 & 0.08	403 (23) & 398 (23)	397 (19) & 402 (23)	385 (27) & 412 (35)	397 (18) & 407 (25)	QTcF
Griffet (2016)^20^	NR	70.9 (13.5)	0.302	0.008	0.026	0.008	403 (27)	393 (22)	394 (21)	394 (21)	QTcF & QTcH
Hadley (2019)^21^	NR	NR	0.63	0.65	NR	NR	NR	NR	NR	NR	QTcF
Huttin (2018)^22^	NR	60 (11)	NR	NR	NR	NR	396 (41)	396 (34)	NR	NR	QTcF
Karjalainen (1994)^23^	39−120	NR	NR	NR	NR	NR	NR	NR	NR	NR	QTcF
Kujanik (2013)^24^	48−90	NR	NR	NR	NR	NR	407 (369−439)	395 (370−425)	NR	393 (367−426)	QTcH
Oo (2016)^25^	NR	NR	0.3	0.01	0.08	0.02	NR	NR	NR	NR	QTcF & “institutional”
Pickham (2016)^26^	<100	NR	0.248	0.004	0.005	0.057	NR	NR	NR	NR	QTcF
Wong (2012)^27^	NR	NR	0.31	0.15	NR	NR	391.2 (26.4)	386.79 (24.5)	386.79 (24.4)	387.52 (24.4)	QTcF

*Note*: *Gervasi (2017) reported results in 13−17 years age group and 18−35 years separately.

Abbreviations: BPM, beats per minute; NR, not reported; QTcB, Bazett; QTcF, Fridericia; QTcFr, Framingham; QTcH, Hodges; SD, standard deviation.

Nine of the 10 studies determined that Fridericia was a preferred method of QT correction in this population as it had the least HR variability and dependence. In all of these studies, QTcF had the lowest *R* ^2^ values and was reported to be most accurate in terms of its ability to remove the effects of HR at all HR ranges.^18^, ^19^, ^20^, ^21^, ^22^, ^23^, ^25^, ^26^, ^27^ Notably, Fridericia was the preferred method proposed by all of the cross‐sectional studies, and no papers specifically discouraged the use of QTcF.^18^, ^19^, ^20^, ^21^, ^22^, ^23^, ^25^, ^26^, ^27^

Additionally, three studies determined that Framingham was not suitable for use for QT correction in athletes and young people,^19^, ^23^, ^25^ and was not specifically recommended by any of the included studies. QTcFr was reported as an alternative to QTcB in two studies, however, it performed worse than Fridericia in both.^18^, ^23^ QTcFr was reported to underestimate QTc at higher HR and overestimate at low HR with significant variability at extremes of HRs in both studies.

Hodges was a preferred alternative in one study, however the authors concluded that the formula remains unreliable due to the observed variability dependent on age and lack of reference range in the literature.^20^ One study identified a novel QT correction algorithm to be better than Bazett (and as good as Fridericia), but this formula has not been included in other studies.^18^

There were insufficient data in the included studies to compare the correlation values of the QTc formula with HRs between studies. The included studies noted different reference ranges for QTc using the different formulae, however, there was no standard reporting of the values to be able to combine this outcome between studies. Studies reported maximum QTc, 95th percentile or median QTc values. No study identified if included participants were diagnosed with LQTS.

## 4 DISCUSSION

### 4.1 Summary of findings

Almost all studies concluded that Bazett was the least reliable method for removing the effect of HR in athletes and young people. QTcB was found to be inaccurate at both high (>90 BPM) and low (<60 BPM) HRs. Alternative QT correction algorithms, most commonly Fridericia, were reported to provide more accurate correction of QT interval at HRs seen in athletes and young people.

### 4.2 Comparison of QT correction algorithms

The Fridericia formula was the preferred algorithm in the vast majority of the included studies. QTcF has been demonstrated to have the most accurate and reliable calculation of the corrected QT interval, particularly at low HRs. This was also supported by a recent study of QT correction in elite Australian cricketers, which was published subsequent to our search date cutoff.^28^ The Fridericia formula is based on a cubed root for correction of the RR interval. The novel derivation, which was performed in the Aihoshi et al. study, identified that a cube root algorithm was the most reliable way of eliminating the inverse relationship between QTc and RR.^18^

Hodges, a linear correction formula, was considered in 7 of the included studies and was only found to be reliable in 1 study.^25^ The findings of the included studies however indicate that in an athletic population, the Fridericia formula is likely to be superior. The Framingham formula, also a linear correction, was assessed in 7 studies but was not the preferred method for correcting for HR in any of the included studies.^19^, ^20^, ^21^, ^23^, ^25^, ^26^, ^27^ It performed better than QTcB in all studies, but was inferior to QTcF.

### 4.3 Measurement of QT intervals

Most of the included studies utilized automated computer algorithms in the assessment of the QTc. Although there are known issues with accuracy and reliability of automatic QTc calculation in athletes due to training‐induced repolarisation abnormalities, it continues to be commonplace as many ECG machines report QTc automatically.^29^, ^30^, ^31^ There are two methods for the determination of the QT interval based on either the threshold or the voltage, which give slightly different values for the QT interval. Another consideration is that manual measurement of the QT interval (even by a cardiologist) may not be reliable, with on study reporting errors of under and over measurement being common.^32^

The automated method of calculation of the QTc may also offer advantages in athletes due to the high frequency of sinus arrhythmia with the subsequent variation in RR interval. The ability of the computer algorithms to measure multiple beats and determine an average may therefore increase the accuracy of this approach. However, marked variation in RR intervals makes the accurate determination of the QTc challenging, and QTc calculation should be avoided in these instances.^29^

### 4.4 The LQTS population

Contrary to the finding that Bazett is a poor formula for correction of the QTc, a recent study compared different correction formulas in patients with confirmed LQTS1 and LQTS2.^33^ This study found that Bazett was superior to Fridericia, Framingham, and Hodges regardless of whether the patients were on or off treatment with beta‐blockers. This finding is at odds with our findings, as well as previous studies which have questioned the reliability of Bazett formula.^29^ It is yet to be determined if patients with LQTS have different RR/QT dynamics compared to healthy controls.

### 4.5 Diagnosis of LQTS in athletes

In general, the diagnosis of LQTS is based upon one of three diagnostic criteria. First, a repeated ECG with a QTcB ≥480 ms, a Long QT or Schwartz score ≥3 or a positive genetic result for a known mutation leading to LQTS.^2^ The Schwartz score in particular mandates the use of the Bazett formula.^34^

With the current athlete screening practices, the diagnosis of LQTS ultimately relies heavily on the resting 12 lead ECG. A problem with the screening of athletes for prolonged QTc is the variation in the QTc even among persons with LQTS. There can be day‐to‐day variations in the QT dependent upon the autonomic status of the patient. The influence of training on genotype‐negative athletes with borderline or slightly abnormal QT intervals should additionally be considered, and there may be a need for reassessment of the diagnosis after a period of detraining.^31^

### 4.6 Setting appropriate QTc thresholds

A phenomenon that is often under‐appreciated is that there are two standard deviation curves that overlap. The upper tail of the QTc for the normal population overlaps with the lower tail of those with LQTS.^35^ The median QTc for persons with LQTS is 460 ms, which falls below the diagnostic range for the condition.^35^ This is well below the diagnostic criteria for LQTS diagnosis in both the clinical guidelines and athlete ECG interpretation guidelines.^4^, ^36^

In the context of screening, where the pretest probability is low, the higher cut‐off scores currently in use are appropriate to rule out LQTS. However, an athlete with a family history of LQTS, unexplained SCD in a family member or a personal history of syncope during exercise has higher pretest probability, which should be considered when measuring the QT interval.

The screening of athletes for a prolonged QTc should therefore only be considered a screening test with further diagnostic approaches required before a final diagnosis and recommendations of limitations or treatment should be applied. Nevertheless, further research is required to establish appropriate reference ranges and determine the diagnostic accuracy of the ECG which is performed in the screening of athletes against a gold standard of the current diagnostic criteria for LQTS.

### 4.7 Limitations

There are several limitations of this systematic review. First, there was no standardized reporting of outcomes for the studies. This limited the ability to report a conclusion other than an indication of how many studies found a particular correction formula to be the preferred method, based on their population inclusion.

The differences in QTc across different formulae included in each study are arguably within limits of variation and further accurate measures would require an invasive electrophysiological study. The combination of correction formulae may introduce measurement bias and new references ranges for QT interval need to be established.

Furthermore, most the studies were from Europe or the United States of America, with participants predominantly of a Caucasian background. This may limit the paper's application to different population groups, as ethnicity is known to affect athlete ECGs.^37^ The information which was provided in the included studies did not allow for the assessment of heterogeneity in the populations or whether there were different subgroups.

QT‐RR hysteresis is a phenomenon not considered by the studies in this review and would affect the length of the QT intervals presented. The phenomenon describes that QT intervals increase during exercise and decrease during recovery.^38^ The state of recovery is unknown in the participants in this study, which could limit the accuracy of QT interval measurement, however most participants are likely to have been at rest without recent exercise.

Not all sports require the same level of physical fitness, which alters the resting HR of individual athletes. Focused demographics of participants limited the types of sports which are being investigated in the reviews, further limiting its application to athletes participating in other sports. Potential variability in the inclusion of participants due to the highly varied interpretation of the term “athlete,” also limits its generalizability.

Finally, these studies were looking at the performance of QT correction algorithms and did not have the diagnosis of LQTS as an outcome.

## 5 CONCLUSIONS

The Bazett method of QT correction is less accurate at lower and higher HRs. Amongst other correction formulae, Fridericia has been shown to be more accurate in these HR ranges and may be preferred to Bazett for QTc calculation in athletes and young people with a tendency toward sinus bradycardia. The differences between the various formulae may have important implications in clinical practice, such as false positives or negatives on screening ECGs. Furthermore, accurate QTc reference values for discrete HRs using alternative algorithms are not well established and require further research. Therefore, while alternative formulae may be marginally more accurate than Bazett in calculating QTc, their clinical utility remains currently limited.

## CONFLICT OF INTEREST STATEMENT

The authors declare no conflict of interest.

## Supporting information

Supporting information.

## Data Availability

All relevant data are within the manuscript and supplementary material.
