# Supplementary material for: Comparison of methods for correcting QT interval in athletes and young people: A systematic review
Source: Clin Cardiol. 2023 Jul 20;46(9):1106–15. doi: 10.1002/clc.24093 (PMC10540007; doi:10.1002/clc.24093)
Supplement: Supplementary file 1 — Supporting information. [file CLC-46-1106-s001.docx]

**Supplementary Appendix A: Search Terms and Search Strategy**

Medline:

| **#** | **Searches** | **Results** |
| --- | --- | --- |
| 1 | exp Athletes/ | 18310 |
| 2 | athlete*.mp. | 66317 |
| 3 | player*.mp. | 71235 |
| 4 | sportsmen.mp. | 1531 |
| 5 | sportsperson*.mp. | 132 |
| 6 | sportswomen.mp. | 148 |
| 7 | exp Young Adult/ | 993352 |
| 8 | exp Adolescent/ | 2180737 |
| 9 | young adult.mp. | 1017590 |
| 10 | adolescent.mp. | 2217476 |
| 11 | youth.mp. | 87352 |
| 12 | pre-participation screening.mp. | 245 |
| 13 | Outpatients/ | 19802 |
| 14 | outpatients.mp. | 65763 |
| 15 | 1 or 2 or 3 or 4 or 5 or 6 or 7 or 8 or 9 or 10 or 11 or 12 or 13 or 14 | 2859806 |
| 16 | electrocardiography/ | 199284 |
| 17 | electrocardiogra*.mp. [mp=title, abstract, original title, name of substance word, subject heading word, floating sub-heading word, keyword heading word, organism supplementary concept word, protocol supplementary concept word, rare disease supplementary concept word, unique identifier, synonyms] | 244810 |
| 18 | ECG.mp. | 70206 |
| 19 | EKG.mp. | 5870 |
| 20 | QTc.mp. | 9286 |
| 21 | QT interval.mp. | 10221 |
| 22 | QT correction formula.mp. | 22 |
| 23 | 20 or 21 or 22 | 16230 |
| 24 | Bazett.mp. | 393 |
| 25 | Hodges.mp. | 525 |
| 26 | Fridericia.mp. | 414 |
| 27 | Framingham.mp. | 10018 |
| 28 | Rautaharju.mp. | 29 |
| 29 | 24 or 25 or 26 or 27 or 28 | 11061 |
| 30 | 15 and 23 and 29 | 172 |

Embase:

| **#** | **Searches** | **Results** |
| --- | --- | --- |
| 1 | elite athlete/ or athlete/ or professional athlete/ | 59949 |
| 2 | athlete*.mp. | 92318 |
| 3 | player*.mp. | 88430 |
| 4 | sportsmen*.mp. | 2830 |
| 5 | sportsperson*.mp. | 169 |
| 6 | sportswomen.mp. | 212 |
| 7 | young adult/ | 463399 |
| 8 | young adult*.mp. | 561616 |
| 9 | adolescent/ | 1683312 |
| 10 | adolescent.mp. | 1728710 |
| 11 | juvenile/ | 56920 |
| 12 | juvenile.mp. | 166689 |
| 13 | outpatient/ | 151133 |
| 14 | pre-participation screening.mp. | 439 |
| 15 | outpatient.mp. | 374638 |
| 16 | 1 or 2 or 3 or 4 or 5 or 6 or 7 or 8 or 9 or 10 or 11 or 12 or 13 or 14 or 15 | 2680403 |
| 17 | electrocardiography/ | 142275 |
| 18 | electrocardiograph*.mp. | 193248 |
| 19 | QT interval/ | 17172 |
| 20 | qt interval.mp. | 24552 |
| 21 | QTc interval/ | 7132 |
| 22 | QTc interval*.mp. | 12158 |
| 23 | QTc.mp. | 18413 |
| 24 | QT.mp. | 56441 |
| 25 | QT correction formula.mp. | 39 |
| 26 | 17 or 18 or 19 or 20 or 21 or 22 or 23 or 24 or 25 | 239629 |
| 27 | Bazetts.mp. | 1046 |
| 28 | Fridericia.mp. | 694 |
| 29 | Hodges.mp. | 798 |
| 30 | Framingham.mp. | 17998 |
| 31 | Rautaharju.mp. | 43 |
| 32 | 27 or 28 or 29 or 30 or 31 | 20189 |
| 33 | 16 and 26 and 32 | 226 |

Scopus:

TITLE-ABS-KEY ( athlete*  OR  player*  OR  sportsmen  OR  sportswomen  OR  sportsperson  OR  youth  OR  adolescent  OR  "young adult"  OR  outpatient  OR  "pre-participation screening" )  AND  TITLE-ABS-KEY ( electrocardiogra*  OR  ecg  OR  ekg  OR  qtc  OR  qt  OR  "QT correction"  OR  "QT correction formula" )  AND  TITLE-ABS-KEY ( bazett  OR  hodges  OR  fridericia  OR  framingham  OR  rautaharju )

SPORTDiscus:

(athlete* OR player* OR sportsmen OR sportswomen OR sportsperson OR youth OR adolescent) AND (electrocardiogra* OR ecg OR ekg OR qtc OR qt) AND (Bazett OR hodges OR fridericia OR framingham OR rautaharju)

**Supplementary Appendix B: Data Extraction Template**


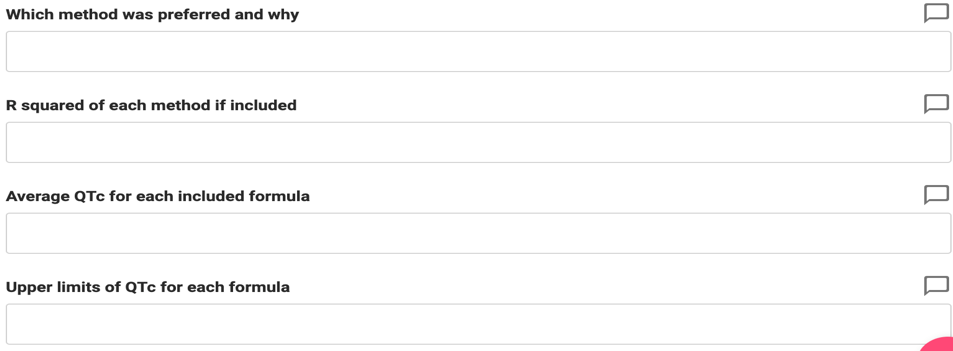

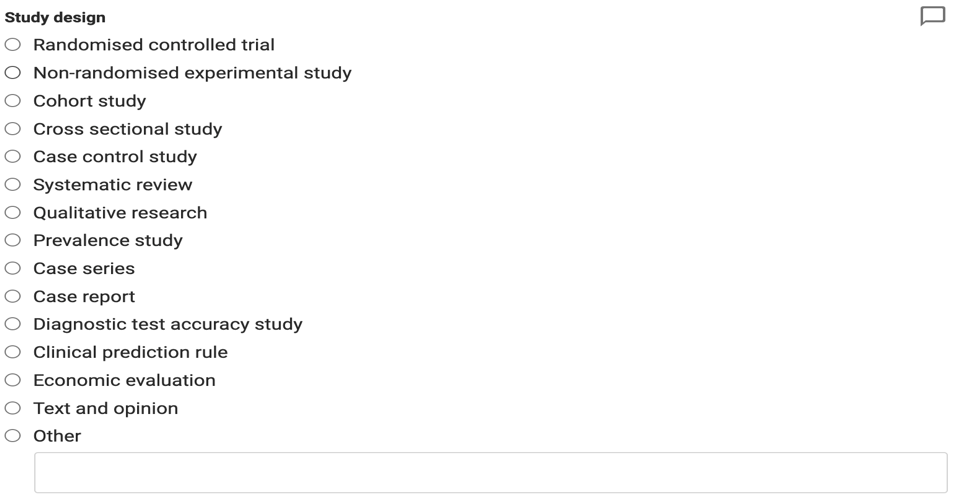

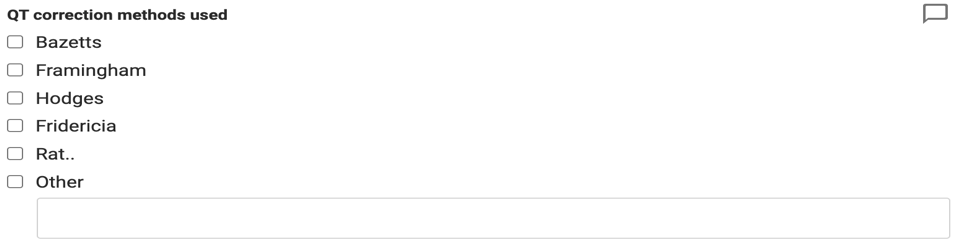

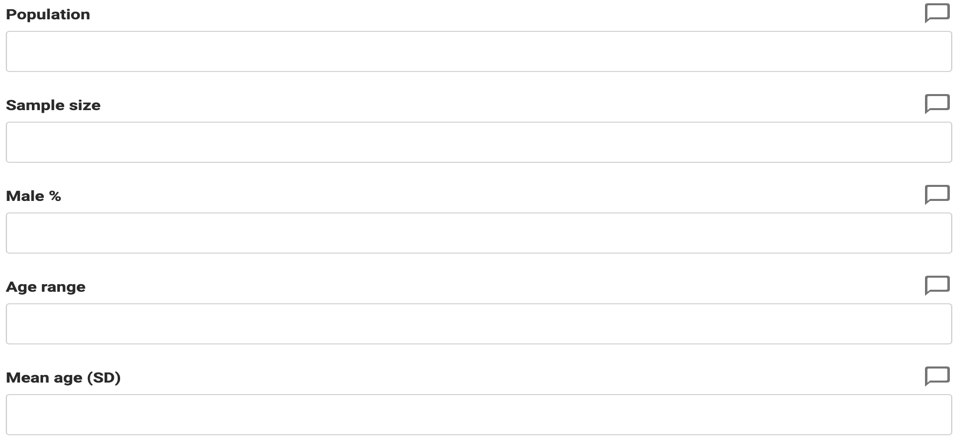

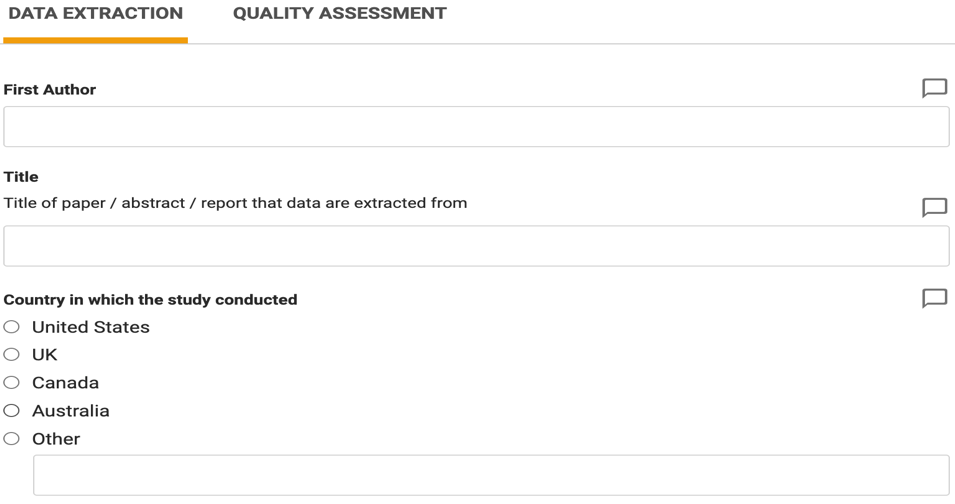


**Supplementary Appendix C: Quality assessment**

| Study ID | 1 | 2 | 3 | 4 | 5 | 6 | 7 | 8 | 9 | 10 | 11 | 12 | 13 | 14 | Final assessment |
| --- | --- | --- | --- | --- | --- | --- | --- | --- | --- | --- | --- | --- | --- | --- | --- |
| Wong 2012 | Yes | Yes | N/A | Yes | No | N/A | N/A | N/A | N/A | N/A | Yes | N/A | N/A | Yes | Fair |
| Pickham 2016 | Yes | Yes | Yes | Yes | No | N/A | N/A | N/A | N/A | N/A | Yes | No | N/A | Yes | Fair |
| Oo 2016 | Yes | No | N/A | N/A | No | N/A | N/A | N/A | N/A | N/A | Yes | N/A | N/A | Yes | Fair |
| Kujanik 2013 | Yes | Yes | N/A | N/A | No | N/A | N/A | N/A | N/A | N/A | N/A | N/A | N/A | N/A | Fair |
| Karjalainen 1994 | Yes | Yes | N/A | Yes | No | N/A | N/A | N/A | N/A | N/A | Yes | N/A | N/A | N/A | Fair |
| Huttin 2018 | Yes | Yes | N/A | Yes | Yes | N/A | N/A | N/A | N/A | N/A | Yes | N/A | No | Yes | Fair |
| Hadley 2019 | Yes | Yes | N/A | Yes | No | N/A | N/A | N/A | N/A | N/A | Yes | N/A | N/A | Yes | Fair |
| Griffet 2016 | Yes | Yes | N/A | N/A | N/A | N/A | N/A | N/A | Yes | No | N/A | N/A | N/A | N/A | Fair |
| Gervasi 2017 | Yes | Yes | N/A | Yes | No | N/A | N/A | N/A | N/A | N/A | Yes | N/A | N/A | Yes | Fair |
| Aihoshi 1995 | Yes | Yes | N/A | Yes | No | N/A | N/A | N/A | N/A | N/A | Yes | N/A | N/A | Yes | Fair |

Key:

Question 1: Was the research question or objective in this paper clearly stated?

Question 2: Was the study population clearly specified and defined?

Question 3: Was the participation rate of eligible persons at least 50%?

Question 4: Were all the subjects selected or recruited from the same or similar populations (including the same time period)? Were inclusion and exclusion criteria for being in the study prespecified and applied uniformly to all participants?

Question 5: Was a sample size justification, power description, or variance and effect estimates provided?

Question 6: For the analyses in this paper, were the exposure(s) of interest measured prior to the outcome(s) being measured?

Question 7: Was the timeframe sufficient so that one could reasonably expect to see an association between exposure and outcome if it existed?

Question 8: For exposures that can vary in amount or level, did the study examine different levels of the exposure as related to the outcome (e.g., categories of exposure, or exposure measured as continuous variable)?

Question 9: Were the exposure measures (independent variables) clearly defined, valid, reliable, and implemented consistently across all study participants?

Question 10: Was the exposure(s) assessed more than once over time?

Question 11: Were the outcome measures (dependent variables) clearly defined, valid, reliable, and implemented consistently across all study participants?

Question 12: Were the outcome assessors blinded to the exposure status of participants?

Question 13: Was loss to follow-up after baseline 20% or less?

Question 14: Were key potential confounding variables measured and adjusted statistically for their impact on the relationship between exposure(s) and outcome(s)?

NA: Not applicable
